# Supplementary material for: Comparative multiomics analysis of cell physiological state after culture in a basket bioreactor
Source: Sci Rep. 2022 Nov 23;12:20161. doi: 10.1038/s41598-022-24687-4 (PMC9686226; doi:10.1038/s41598-022-24687-4)

23666063.1:13960981:13961064:-@NW_023666063.1:13960697:13961064:-@NW_023666063.1:13960697:1396

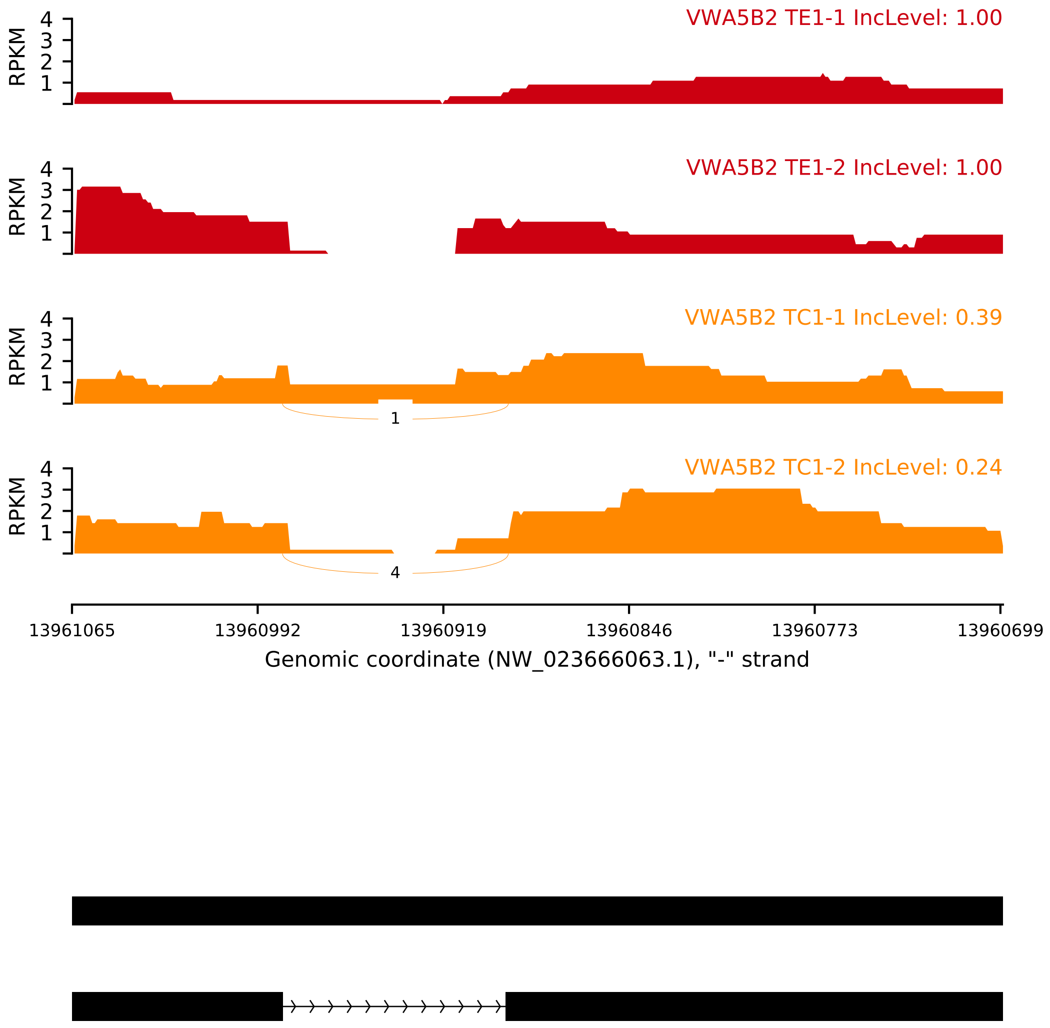

Supplement: Supplementary file 1 — Supplementary Information 1. [file 41598_2022_24687_MOESM1_ESM.zip › raw data/Transcriptomic raw data/7.AS/2.dasplot/TE1vsTC1/RI/14_VWA5B2_NW_023666063.1_13960981_13961064_-@NW_023666063.1_13960697_13961064_-@NW_023666063.1_13960697_13960893_-.pdf]
